# The effect of repetitive transcranial magnetic stimulation on sleep quality in patients with more than mild depressive mood: a systematic review and meta-analysis

**DOI:** 10.3389/fpsyt.2025.1511930

**Published:** 2025-02-06

**Authors:** Yu’ang Liu, Silang Huang, Xinxin Zhang, Huangying Liao, Weiguo Liu, Zhi Zhang, Xianhui Qu, Ziwen Wang

**Affiliations:** ^1^ College of Physical Education and Health, Guangxi Normal University, Guilin, Guangxi, China; ^2^ College of Education, Guilin University, Guilin, Guangxi, China; ^3^ College of Foreign Languages, Guangxi Normal University, Guilin, Guangxi, China

**Keywords:** repetitive transcranial magnetic stimulation, depressive moods, sleep quality, meta-analysis, review

## Abstract

**Background:**

The purpose of this study was to systematically evaluate the efficacy of repetitive transcranial magnetic stimulation (rTMS) therapy on sleep quality in patients with more than mild depressive mood.

**Methods:**

Randomized controlled trials in PubMed, Cochrane Library, Web of Science, Embase, Scopus, and ScienceDirect on rTMS to improve sleep quality in patients with more than mild depressive mood up to September 2023 were searched. A meta-analysis was performed using RevMan 5.4 and Stata 17.0 software.

**Results:**

A total of 11 studies, which involved 548 patients, were included. After rTMS treatment, the effect on sleep quality improvement in patients with more than mild depressive mood was better in the experimental group than in the control group [*I* ^2^ = 53%, mean difference (MD) = −2.27, 95%CI = −2.97 to −1.57, *p* < 0.00001]. The results of the subgroup analyses showed that, in terms of stimulation frequency, compared with the 5-Hz and 10-Hz groups, the treatment effect of the 1-Hz group was better (*I* ^2^ = 32%, MD = −2.69, 95%CI = −3.78 to −1.60, *p* < 0.00001). In terms of treatment duration, compared with the 2-week and 4-week groups, the group with more than 4 weeks of treatment had better treatment outcomes (*I* ^2^ = 0%, MD = −2.81, 95%CI = −3.22 to −2.40, *p* < 0.00001). In terms of whether combination therapy was used or not, compared with the combination therapy group (*I* ^2^ = 29%, MD = −1.39, 95%CI = −2.30 to −0.48, *p* = 0.003), the non-combination therapy group had a better treatment effect (*I* ^2^ = 0%, MD = −2.93, 95%CI = −3.36 to −2.50, *p* < 0.00001).

**Conclusion:**

rTMS significantly improves sleep quality in patients with more than mild depression. Subgroup analyses showed that the group using the 1-Hz stimulation frequency, the group with more than 4 weeks of treatment time, and the group with rTMS alone had better efficacy in treating the sleep quality of patients with more than mild depressive mood using rTMS, with the use of combination treatment or not being the main source of heterogeneity.

**Systematic review registration:**

https://www.crd.york.ac.uk/prospero/, identifier CRD42023467971

## 1 Introduction

Sleep disorders and depressive disorders have become major challenges for humanity and modern medicine. A study of more than 2,000 participants showed a 32% incidence of sleep disorders (1). Another study showed that more than 350 million people worldwide now suffer from depressive disorders (2). A study also showed that approximately 61.8% of people with depressive disorders had sleep disorders as the primary manifestation (3).

The close relationship between sleep quality and depression is complex, although it is not clear whether there is an etiologic relationship between sleep quality and depression. However, sleep quality is highly correlated with depression severity (4). A number of studies have shown significantly elevated levels of depression and anxiety symptoms in patients with circadian rhythm sleep–wake disorder (5). Furthermore, an improved sleep structure may have a positive effect on depression (6). Research also suggests that sleep quality can predict future depression (7). In addition, poor sleep can lead to mental health problems (8, 9). The reason for this may be that sleep interacts with mental diseases and physiological disorders, leading to the deterioration of human mental health (10–12). Chronic low sleep quality could exacerbate depression, thus creating a vicious cycle; therefore, the improvement of sleep quality is important for depression relief.

The treatment of sleep disorders is generally based on drug therapy; however, there are some issues such as drug dependence and cognitive impairment, which place certain limitations on its clinical application (13). In recent years, repetitive transcranial magnetic stimulation (rTMS) has received increasing attention from medical professionals, indicating its effectiveness in improving sleep quality or sleep disorders in patients with depressive mood (14, 15). It can not only cause excitatory changes in the cerebral cortex, a variety of neurotransmitter changes, but also improve brain function and blood circulation and relieve bad mood. Changes in these factors can lead to changes in sleep quality. Low-frequency (1 Hz) trans-frequency magnetic stimulation inhibits nerve cell metabolism and decreases cortical excitability, whereas high-frequency (>1 Hz) trans-frequency magnetic stimulation promotes nerve cell metabolism and cortical excitability (16).

Although rTMS has been approved for the treatment of depression, evaluation of the efficacy of treatment has mainly utilized the Hamilton Depression Inventory and the Hamilton Anxiety Inventory. There is a lack of evaluation of sleep quality. On the other hand, patients with failed back surgery syndrome, patients with Parkinson’s, and those with fibromyalgia, among others, are often accompanied with more than mild depressive mood and sleep disorders and have not been comprehensively included in the study, leading to the use of only a single subject in the previous study. Hence, the results of the study have low reliability and validity, and the conclusions are not comprehensive enough. Therefore, the aim of this study was to systematically evaluate the efficacy of rTMS on sleep quality in patients with more than mild depressive mood based on existing studies. Patients with more than mild depressive mood are not necessarily depressed patients, but are diagnosed as having a certain depressive mood in the depression scale test.

## 2 Materials and methods

This review was registered (identifier: CRD42023467971) in the International Prospective Register of Systematic Reviews (PROSPERO) and complied with the Preferred Reporting Items for Systematic Reviews and Meta-Analyses (PRISMA) statement.

### 2.1 Study search and selection

We searched the Cochrane Library, PubMed, Web of Science, Scopus, Embase, and ScienceDirect databases for randomized controlled trials on the effect of rTMS on sleep quality in patients with more than mild depressive mood, published in full text from the time of the construction of the library to September 2023, using a combination of subject headings and free-text headings via a computer network. The language is in English only. The English search terms mainly included “Transcranial Magnetic Stimulation,” “Depression,” “Sleep quality,” and “Dyssomnias.”

The EndNote X9 software was used to remove duplicates from the search, and then two reviewers independently read the titles and abstracts of the articles to establish eligibility for inclusion. Studies that failed to meet the inclusion criteria were not reviewed further. Those that could not be excluded were retrieved, and two reviewers (YL and XZ) assessed the whole text. The authors were contacted via e-mail when data validation or more information was required. Disagreements or ambiguities were resolved through discussion with a third reviewer (SH).

### 2.2 Inclusion criteria

The inclusion criteria were as follows:

1. Subjects in the experimental and control groups were required to have a diagnosis of more than mild depressive mood and sleep quality disorders: Hamilton Depression Index 17 scores greater than 7 (17), Depression Self-Assessment Index scores greater than 60, Beck’s Depression Index scores greater than 20, and Pittsburgh Sleep Quality Index scores greater than 7 (18).
2. The study design is a randomized controlled trial or a clinical trial.
3. The outcome metrics of the experimental and control groups contained data related to the evaluation of sleep quality.
4. The rTMS intervention is the only variable for the experimental and control groups.
5. There are no statistically significant differences in the pretreatment general information and the baseline characteristics between the experimental and control groups.

### 2.3 Literature exclusion criteria

The exclusion criteria were as follows:

1. There is no control group in the experiment.
2. There is no report on the baseline that does not report the depression emotional scores and the sleep quality scores or does not report the sleep quality score at the end point.
3. Reviews, dissertations, and conference papers.
4. Both the experimental and control groups used rTMS, but with different stimulation parameters.
5. The sleep quality outcome measures included only the sleep disorder component of the seven components of the Pittsburgh Sleep Quality Index Scale.

### 2.4 Types of outcome measures

The Hamilton Depression Scale, Beck’s Depression Inventory, Depression Self-Rating Scale, and Pittsburgh Sleep Quality (sleep quality was measured by the Pittsburg Sleep Quality Scale) were used.

### 2.5 Data extraction and quality assessment

The following data were obtained from the included studies: information on the first author, year of publication, basic information of the experimental subjects, type of intervention, stimulation time, stimulation parameters, follow-up, country, duration of the experiment, the Depression Index, and the Pittsburgh Sleep Quality Index.

The quality of the included literature was also evaluated independently by two investigators using the Cochrane Collaboration’s risk-of-bias guidelines. In the case of a disagreement, a third investigator was called in to discuss the issue together until a consensus was reached. The evaluation measures included: 1) random sequence generation; 2) allocation blinding; 3) blinding of participants and personnel; 4) blinding of outcome assessors; 2) incomplete ending data; 6) selective reporting of results; and 7) other offsets. A trial was classified as having a “low risk of bias” (“+”) if it was judged to have a low risk of bias for all domains for this result. If it was assessed to raise some concerns in at least one area for this result, but not in any other, then it was categorized as having a “high risk of bias” (“−”). A trial was categorized as having an “uncertain risk of bias” (“?”) if it was considered to have concerns for several domains in a manner that considerably decreases confidence in the results. Discrepancies in the evaluation of the two assessors (YL and ZZ) were resolved through discussion with a third reviewer (WL).

### 2.6 Statistical analysis

All of the outcome indicators were the continuous variables in this study, and the mean difference (MD) or the standardized mean difference (SMD) with 95% confidence interval (95%CI) was used as the effect size to analyze these studies. MD was used when the studies were measured in the same manner or in the same units; otherwise, SMD was used. The meta-analysis produced effect sizes, which are statistically standardized representations of the quantitative findings of each study. These were calculated based on the mean pre–post change in the treatment group minus the mean pre–post change in the comparison group, divided by the pooled pretest standard deviation. Meta-analysis was performed using RevMan 5.4 and Stata 17.0 software. Heterogeneity was quantified using the *I* ^2^ statistic, and a fixed effects model was used when there was no statistically significant difference in the heterogeneity test (*I* ^2^ < 50%, *p* > 0.05); otherwise, a random effects model was used. Subgroup analyses and sensitivity analyses were performed to examine the sources of heterogeneity for the outcome indicators where heterogeneity existed. Publication bias was assessed using funnel plots and Egger’s asymmetry test for the outcome indicators with >10 included studies. All statistical significance levels were set at *α* = 0.05.

### 2.7 Statement of the ethics committee

No experimental studies on humans and animals were conducted in this study, only a meta-analysis of original studies. Ethics committee approval was therefore not required.

## 3 Results

### 3.1 Search results

**Figure 1:**
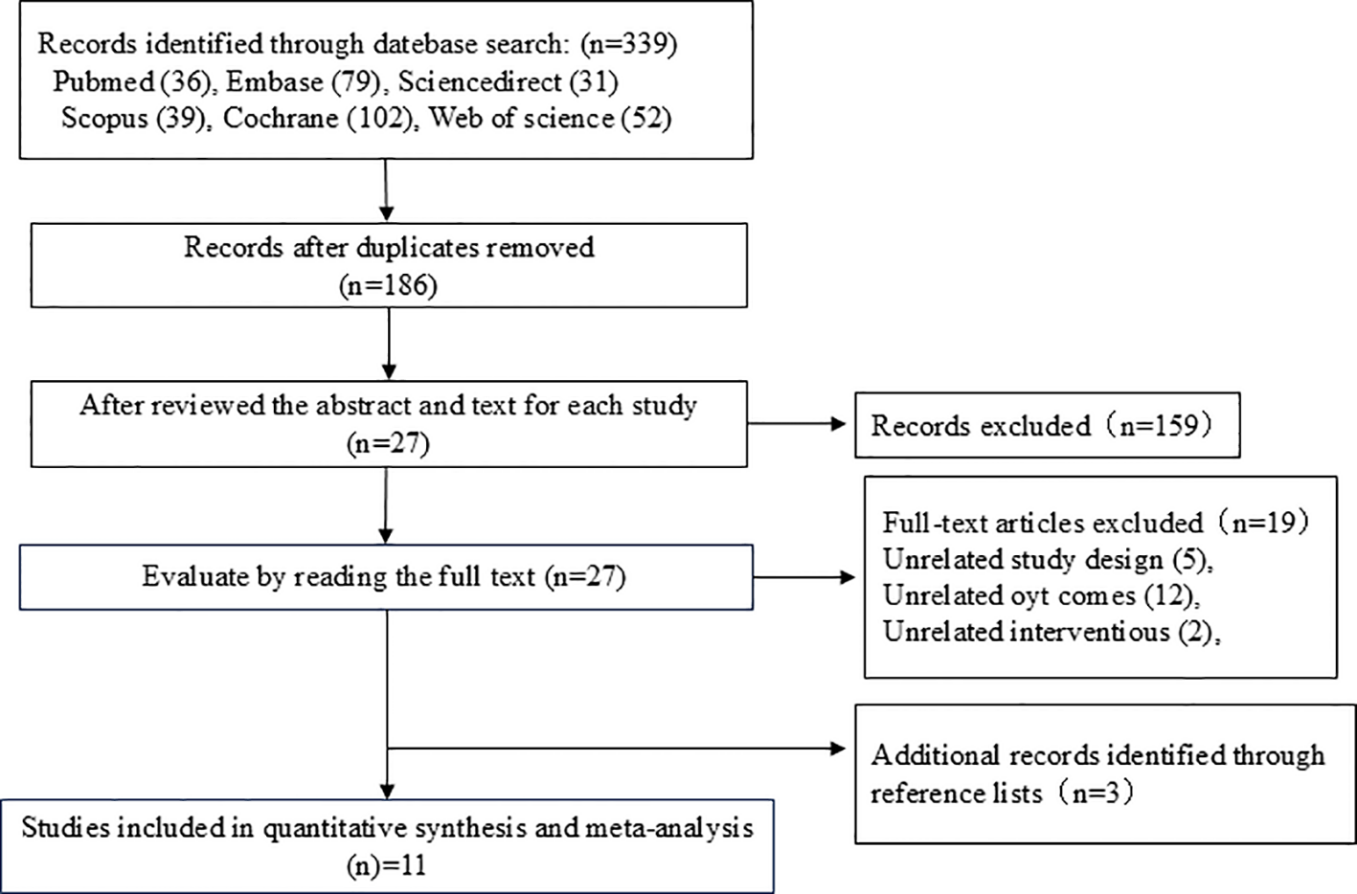
Literature screening flowchart.

A total of 339 pieces of literature were obtained through the database search, with 186 of these remaining after the removal of duplicates using EndNote. After the initial screening by reading the titles and abstracts of the studies, three researchers excluded 159 irrelevant papers based on the inclusion and exclusion criteria. Of the remaining 27 studies, 19 studies that did not meet the inclusion criteria were excluded after reading the full text and rescreening. An additional three studies found in the references that met the inclusion criteria were included. A total of 11 studies were finally included in the meta-analysis. A flowchart of the literature screening process is shown in **Figure 1**.

### 3.2 Participant characteristics

A total of 548 patients from the 11 studies (19–29) were included in this study, of which 287 patients were in the experimental group and 261 patients were in the control group. The basic characteristics of the included studies are shown in **Table 1**. The average age of the patients included in the studies ranged from 33 to 61 years. Six of the studies had patients with mild depression (21, 23, 24, 26, 28, 29), four studies had patients with moderate depression (19, 20, 22, 27), and one study had patients with severe depression (25). In all of the studies, the Pittsburgh Sleep Quality Index was rated as mildly sleep-disordered. The detailed characteristics of each included study are shown in **Table 1**.

**Table 1:** Detailed characteristics of each included study.

Reference	Sample size	Sex (M/F)	Age (mean ± SD)	Depression index	Pittsburgh Sleep Quality Index	Country
Bursali C. et al., 2019 (19)	E: 10	7/3	48.20 ± 9.53	20.1 ± 12.3^a^	9.5 ± 4.38	Turkey
	C: 10	7/3	54.40 ± 10.04	19.8 ± 12.19^a^	9.3 ± 6.34	
Duan H. R. et al., 2023 (20)	E: 23	19/4	58.30 ± 13.06	19.04 ± 2.16^b^	10.87 ± 1.87	China
	C: 24	20/4	53.63 ± 13.01	19.13 ± 3.05^b^	11.79 ± 2.54	
Garza-Villarreal E. A. et al., 2021 (21)	E: 24	20/4	35.8 ± 7.1	13.8 ± 7.5^b^	10.8 ± 4.2	Mexico
	C: 20	18/2	33.3 ± 8.7	13.9 ± 10.2^b^	10.7 ± 5.1	
Guinot M. et al., 2021 (22)	E: 18	0/18	46.5 ± 10.4	25.6 ± 11.2^a^	13.9 ± 4.0	France
	C: 19	4/15	42.8 ± 8.8	23.5 ± 11.1^a^	12.2 ± 3.5	
Huang Z. et al., 2018 (23)	E: 18	9/9	44.94 ± 11.64	13.17 ± 4.30^b^	12.61 ± 2.85	China
	C: 18	9/9	45.22 ± 10.85	13.06 ± 2.90^b^	13.06 ± 4.26	
Li H. et al., 2021 (24)	E: 45	NR	NR	62.95 ± 3.83^c^	10.57 ± 2.04	China
	C: 30	NR	NR	61.75 ± 4.30^c^	11.07 ± 1.72	
Li N. et al., 2013 (25)	E: 30	18/12	34 ± 7	46.1 ± 7.6^a^	9.4 ± 4.3	China
	C: 29	15/14	33 ± 9	46.4 ± 7.5^a^	9.4 ± 5.4	
Lin J. et al., 2019 (26)	E: 40	40/0	33.95 ± 7.13	63.80 ± 4.45^c^	10.95 ± 1.34	China
	C: 40	40/0	34.5 ± 8.54	60.60 ± 5.33^c^	12.78 ± 1.25	
Pu Z. et al., 2023 (27)	E: 42	NR	35.24 ± 5.12	22.82 ± 6.39^b^	14.62 ± 3.84	China
	C: 40	NR	33.97 ± 4.74	21.45 ± 6.56^b^	14.24 ± 3.67	
Zhou K. et al., 2021 (28)	E: 18	6/12	56.77 ± 9.57	13.00 ± 5.98^b^	14.62 ± 3.31	China
	C: 17	7/11	53.82 ± 10.31	12.60 ± 3.87^b^	12.00 ± 3.26	
Zhuang S. et al., 2020 (29)	E: 19	8/11	60.58 ± 9.21	13.26 ± 6.90^b^	9.63 ± 4.87	China
	C: 14	7/7	61.57 ± 13.25	15.86 ± 7.12^b^	7.57 ± 3.25	

*E*, experimental group; *C*, control group; *M*. male; *F*, female.

a Beck’s Depression Index.

b Hamilton Depression Index.

c Depression Self-Assessment Index.

### 3.3 Intervention protocols

Details of the intervention parameters in the included studies are shown in **Table 2**. Five studies used rTMS alone (19, 21, 23, 26, 29), while six studies used rTMS in combination with therapy (20, 22, 24, 25, 27, 28). Four studies used a stimulation frequency of 1 Hz (23, 25, 28, 29), two studies used 5 Hz (19, 21), five studies used 10 Hz (20, 22, 24, 26, 27), five studies used stimulus intensities below 100% (19, 20, 22, 23, 25), and six studies had stimulus intensities above 100% (21, 24, 26–29). Six studies had stimulus locations in the left dorsolateral prefrontal cortex (L-DLPFC) (20, 21, 24, 26–28), while three studies had stimulus locations in the right DLPFC (R-DLPFC) (23, 25, 29). Interestingly, these studies all used low-frequency rTMS. One study did not report the stimulation location (22), and one study used M1 as the stimulation location (19). Seven studies used an F8 coil type (19, 21, 23–25, 28, 29), one study used a C coil (24), while three studies did not report the coil type used (20, 22, 27). Four studies used a 2-week treatment duration (19, 21, 23, 29), two studies used a 4-week treatment duration (20, 25), and five studies used more than 4 weeks of treatment (22, 24, 26–28). Four studies conducted follow-ups (19, 20, 23, 29), while seven studies did not (21, 22, 24–28). The specific parameters of the rTMS interventions are shown in **Table 2**.

**Table 2:** Specific parameters of the repetitive transcranial magnetic stimulation (rTMS) interventions.

Reference	Intervention type	Stimulation parameters				Total duration of treatment (weeks)	Follow-up
		Frequency (Hz)	Intensity	Site	Coil type		
Bursali C. et al., 2021 (19)	rTMS	5	70% RMT	NR	F8	2	Yes (1 month)
Duan H. R. et al., 2023 (20)	rTMS+MBSR	10	80% RMT	L-DLPFC	NR	4	Yes (8 weeks)
Garza-Villarreal E. A. et al., 2021 (21)	rTMS	5	100% RMT	L-DLPFC	F8	2	No
Guinot M. et al., 2021 (22)	rTMS+MT	10	80% AMT	M1	NR	14	No
Huang Z. et al., 2018 (23)	rTMS	1	90% RMT	R-DLPFC	F8	2	Yes (1 month)
Li H. et al., 2021 (24)	rTMS+HIIT	10	100% RMT	L-DLPFC	C	6	No
Li N. et al., 2013 (25)	rTMS+Drug	1	80% RMT	R-DLPFC	F8	4	No
Lin J. et al., 2019 (26)	rTMS	10	100% RMT	L-DLPFC	F8	6	No
Pu Z. et al., 2023 (27)	rTMS+Drug	10	120% RMT	L-DLPFC	NR	8	No
Zhou K. et al., 2021 (28)	rTMS+Drug	1	100% RMT	L-DLPFC	F8	6	No
Zhuang S. et al., 2020 (29)	rTMS	1	110% RMT	R-DLPFC	F8	2	Yes (2 weeks)

*rTMS*, repetitive transcranial magnetic stimulation; *MBSR*, mindfulness-based stress reduction; *HIIT*, high-intensity interval training; *MT*, multicomponent therapy; *RMT*, resting motor threshold; *AMT*, active motor threshold; *L*, left; *R*, right; *DLPFC*, dorsolateral prefrontal cortex (primary motor cortex); *M1*, dominant thenar area; *C*, circular.

### 3.4 Methodological quality assessment

**Figure 2:**
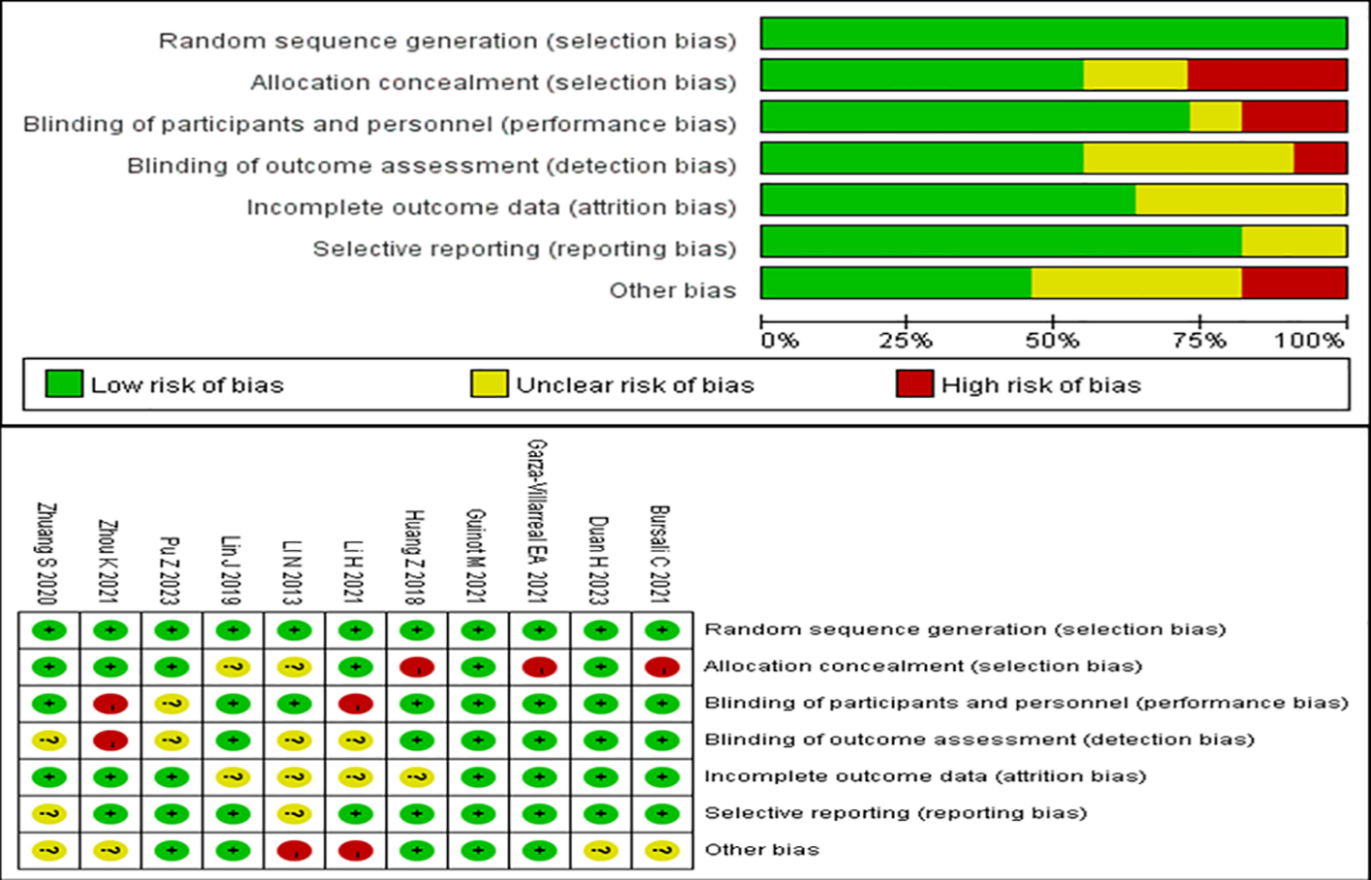
Risk of bias summary.

The methodological quality of all included studies was evaluated using the Cochrane Collaboration’s techniques for assessing bias risk. All of the included trials described randomized allocation and were assessed to have low risk in this field. In allocation blinding, six studies were classified as having a low risk (20, 22, 24, 27–29), while three studies were classified as having a high risk (19, 21, 23). Two studies were evaluated to have an uncertain risk (25, 26). In the blinding of participants and personnel, eight studies were assessed to have a low risk (19–23, 25, 26, 29), with only one study assessed as having an unclear risk (27). The rest of the studies were assessed to have a high risk. In the blinding of outcome assessment, six studies were classified to have a low risk (19–23, 26), with only one study assessed as having a high risk (28). The rest of the studies had an unclear risk. For incomplete outcome data, seven studies were classified to have a low risk (19–22, 27–29), with the rest of the studies having an unclear risk. With regard to selective reporting bias, nine studies were considered to have a low risk (19–24, 26–28), while the remaining studies were considered to have an unclear risk. With regard to other biases, five studies were assessed to have a low risk of other biases (21–23, 26, 27), four studies were assessed to have an unclear risk (19, 20, 28, 29), and two studies were assessed to have a high risk (24, 25). These results are summarized in **Figure 2**.

### 3.5 Meta-analysis results

#### 3.5.1 Meta-analysis of repetitive transcranial magnetic stimulation on sleep quality in patients with more than mild depressive mood

**Figure 3:**
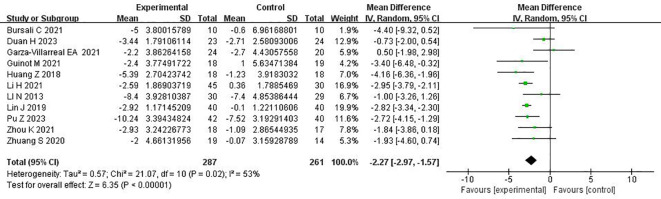
Meta-analysis of repetitive transcranial magnetic stimulation therapy on sleep quality in patients with more than mild depressive mood.

The 11 randomized controlled trials explored the Pittsburgh Sleep Quality Index using a random effects model, with the combined results showing *I* ^2^ = 53%. The rhombus did not intersect with the straight line, and the effector MD mainly fell on the left side of the straight line, indicating that the Pittsburgh Sleep Quality Index was lower in the experimental group than in the control group. As *p* < 0.00001, the experimental group of rTMS for the treatment of patients with more than mild depressive mood was significantly better than the control group in terms of improvement of sleep quality effect (MD = −2.27, 95%CI = −2.97 to −1.57, *p* < 0.00001). The meta-analysis of rTMS on sleep quality in patients with more than mild depressive mood is shown in **Figure 3**.

#### 3.5.2 Subgroup analyses of the effect of repetitive transcranial magnetic stimulation therapy on sleep quality in patients with more than mild depressive mood

For the subgroup analysis of each group, the studies were divided into the 1-Hz, 5-Hz, and 10-Hz groups according to the inclusion of the study stimulation frequency; into the 2-week, 4-week, and more than 4-week groups according to the inclusion of the study treatment time; and into the combination treatment and non-combination treatment groups according to whether a combination treatment was administered. Using a random effects model, the combined results showed that the effect of rTMS therapy on the improvement of sleep quality in patients with more than mild depressive mood was better in the experimental group than in the control group. The results of the subgroup analyses showed that, in terms of stimulation frequency, the treatment effect of the 1-Hz group (*I* ^2^ = 32%, MD = −2.69, 95%CI = −3.78 to −1.60, *p* < 0.00001) was better than that of the 5-Hz group (*I* ^2^ = 67%, MD = −1.47, 95%CI = −6.18 to −3.24, *p* = 0.54) and the 10-Hz group (*I* ^2^ = 59%, MD = −2.22, 95%CI = −3.19 to −1.260, *p* < 0.00001). In terms of treatment duration, compared with the 2-week group (*I* ^2^ = 64%, MD = −2.28, 95%CI = −4.67 to −0.10, *p* = 0.06) and the 4-week group (*I* ^2^ = 0%, MD = −0.79, 95%CI = −1.90 to −0.31, *p* = 0.16), the group of more than 4 weeks (*I* ^2^ = 0%, MD = −2.81, 95%CI = −3.22 to −2.40, *p* < 0.00001) had better treatment outcomes. In terms of whether combination therapy was given, the non-combination treatment group (*I^2^* = 0%, MD = −2.93, 95%CI = −3.36 to −2.50, *p* < 0.00001) had better outcomes than the combination treatment group (*I* ^2^ = 29%, MD = −1.39, 95%CI = −2.30 to −0.48, *p* = 0.003). Due to the combination treatment (*I* ^2^ = 29%, MD = −1.39, 95%CI = −2.30 to −0.48, *p* < 0.00001) and non-combination treatment groups (*I* ^2^ = 0%, MD = −2.93, 95%CI = −3.36 to −2.50, *p* < 0.00001) both showing heterogeneity (*I* ^2^ < 50%; the total outcome was *I* ^2^ > 50%), whether or not combination treatment was used was the main source of heterogeneity in the quality of sleep in depressed patients with rTMS treatment in this study. The subgroup analysis of the effect of rTMS on sleep quality in patients with more than mild depressive mood is shown in **Table 3**.

**Table 3:** Subgroup analysis of the effect of repetitive transcranial magnetic stimulation.

Subgroup analysis		*I* ^2^ (%)	MD	95%CI	p
Stimulation frequency subgroup	1-Hz group	32	−2.69	−3.78 to −1.60	<0.00001
	5-Hz group	67	−1.47	−6.18 to 3.24	0.54
	10-Hz+ group	59	−2.22	−3.19 to −1.26	<0.00001
Treatment time subgroup	2-week group	64	−2.28	−4.67 to −0.10	0.06
	4-week group	0	−0.79	−1.90 to 0.31	0.16
	4-week+ group	0	−2.81	−3.22 to −2.40	<0.00001
Combination treatment/(or not) group	Combination group	29	−1.39	−2.30 to −0.48	0.003
	Non-combination group	0	−2.93	−3.36 to −2.50	<0.00001

### 3.6 Sensitivity analysis

The results of this study were moderately heterogeneous. The sensitivity analysis was carried out using the item-by-item deletion method. After deleting one study (20) through sensitivity analysis, the intervention was rTMS combined with psychotherapy. The other studies did not use rTMS combined with psychotherapy. The combined results of the other studies showed a significant reduction in heterogeneity (*I* ^2^ = 28%), but there was no significant difference in the overall combined results (MD = −2.58, 95%CI = −3.17 to −1.99, *p* < 0.00001), demonstrating that the findings of this study are more reliable.

### 3.7 Publication bias test

**Figure 4:**
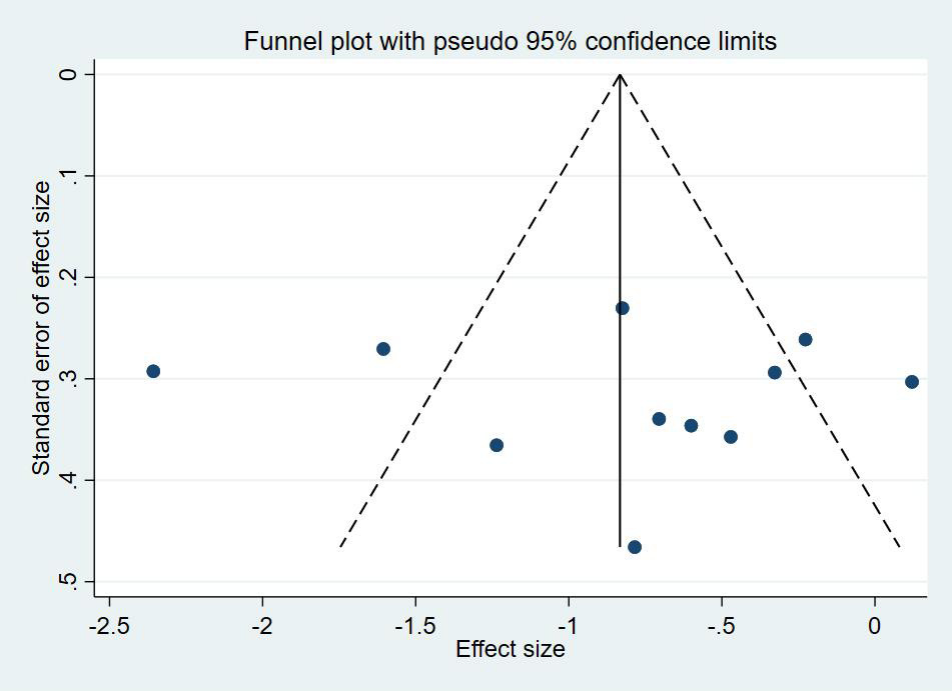
Funnel plots of repetitive transcranial magnetic stimulation on sleep quality in patients with more than mild depressive mood.

The funnel plots (**Figure 4**) and the Egger’s asymmetry test for sleep quality, an outcome metric including more than 10 studies, showed no publication bias (*p* = 0.884).

## 4 Discussion

The aim of this study was to systematically evaluate the efficacy of rTMS for the treatment of sleep quality in patients with more than mild depressive mood. A total of 11 studies involving 548 patients were included in this study, and the results demonstrated that rTMS could significantly improve the sleep quality of patients with more than mild depressive mood. In addition, this study analyzed subgroups in terms of three dimensions: the duration of treatment, the frequency of stimulation, and whether or not combination treatments were used. The results of the subgroup analyses showed that the 1-Hz stimulation frequency, treatment duration of more than 4 weeks, and non-combination treatment had higher reliability for the efficacy of rTMS for the treatment of sleep quality in patients with more than mildly depressive mood and that whether or not the treatment was in the form of a combination was the main source of heterogeneity.

rTMS is a painless and noninvasive green treatment method that has emerged in recent years, and the Pittsburgh Sleep Quality Scale is a valid scale for the evaluation of sleep quality (18). The results of this study showed that rTMS treatment significantly improved the sleep quality of patients with more than mild depression, which is consistent with the results of previous studies (30, 31) showing that low-frequency rTMS can improve sleep quality. The neural mechanism of rTMS to improve sleep quality can be explained as follows: Firstly, low-frequency rTMS can affect the release of neurotransmitters, such as 5-hydroxytryptamine and gamma-amo-butyric acid, and the secretion of melatonin (32, 33), which play an important role in sleep regulation. Secondly, low-frequency rTMS can inhibit the excitability of the cerebral cortex, decrease the activity of neurons, slow down the nerve conduction speed, reduce the synaptic connection of the brain stem reticular structure, and inhibit the function of the upstream brain stem reticular agonist system, thus improving the sleep structure of patients and increasing their non-REM sleep (34). Finally, rTMS can also affect neural networks by increasing synaptic plasticity, affecting signaling pathways, and enhancing gene transcription (35), thereby improving sleep quality.

In addition, the subgroup analysis results showed better sleep quality in patients with more than mild depressive mood treated with rTMS using the 1-Hz stimulation frequency, 4 weeks or more treatment time, and non-combination treatment. In terms of stimulation frequency, low-frequency rTMS mainly affects the inhibitory state of the brain more, thus contributing to the improvement of sleep quality. On the other hand, high-frequency rTMS mainly increases the excitatory state of the brain, which is not conducive to the improvement of sleep quality, but has the potential to improve the quality of sleep by improving the depressive mood of the patient (36). In terms of treatment duration, on the one hand, the brain needs a longer time to adapt to the new stimulation pattern and establish a new biological clock and sleep habits; on the other hand, the improvement of sleep quality may be accompanied by the improvement of depressive mood (37). Therefore, more than 4 weeks of treatment duration is more effective. In terms of whether to use combination treatment, non-combination therapy had a better effect relative to combination treatment. This may be due to the patients included in the study being more diverse and their conditions not consistent; moreover, whether to use combination therapy needs to be determined according to the symptoms and the needs of patients.

This study has some limitations. It was limited by the small sample size. Moreover, the evaluation indices of the treatment effect were not comprehensive, and EEG and polysomnography, as well as the combination of imaging examinations, were not used for comprehensive evaluation. Therefore, a large-sample clinical study that integrates multiple evaluation systems with a more detailed design is needed for a comprehensive observation.

## 5 Conclusions

rTMS significantly improves sleep quality in patients with more than mild depression. Subgroup analyses showed that the group using a 1-Hz stimulation frequency, the group with more than 4 weeks of treatment time, and the group that used rTMS alone had better efficacy in treating the sleep quality of patients with more than mild depressive mood with rTMS, with the use (or not) of combination treatment being the main source of heterogeneity.

## Data Availability

The original contributions presented in the study are included in the article/supplementary material, further inquiries can be directed to the corresponding author/s.
